# Determinants of dietary behaviour in wheelchair users with spinal cord injury or lower limb amputation: Perspectives of rehabilitation professionals and wheelchair users

**DOI:** 10.1371/journal.pone.0228465

**Published:** 2020-01-31

**Authors:** Jasmijn F. M. Holla, Lizanne E. van den Akker, Tessa Dadema, Sonja de Groot, Michael Tieland, Peter J. M. Weijs, Marije Deutekom, on behalf of the WHEELS-study group

**Affiliations:** 1 Faculty of Health, Sports and Social Work, Inholland University of Applied Sciences, Haarlem, The Netherlands; 2 Amsterdam Rehabilitation Research Centre, Reade, Amsterdam, The Netherlands; 3 Faculty of Health, Amsterdam University of Applied Sciences, Amsterdam, The Netherlands; 4 University Medical Centre Groningen, Centre for Human Movement Sciences, University of Groningen, Groningen, The Netherlands; 5 Faculty of Sports and Nutrition, Amsterdam University of Applied Sciences, Amsterdam, The Netherlands; 6 Department of Nutrition and Dietetics, Amsterdam University Medical Centres, VU University, Amsterdam, The Netherlands; KITE-Toronto Rehabilitation Institute-University Health Network, CANADA

## Abstract

**Objective:**

This study aims to identify determinants of dietary behaviour in wheelchair users with spinal cord injury or lower limb amputation, from the perspectives of both wheelchair users and rehabilitation professionals. The findings should contribute to the field of health promotion programs for wheelchair users.

**Methods:**

Five focus groups were held with wheelchair users (n = 25), and two with rehabilitation professionals (n = 11). A thematic approach was used for data analysis in which the determinants were categorized using an integrated International Classification of Functioning, Disability and Health and Attitude, Social influence and self-Efficacy model.

**Results:**

Reported personal factors influencing dietary behaviour in wheelchair users were knowledge, boredom, fatigue, stage of life, habits, appetite, self-control, multiple lifestyle problems, intrinsic motivation, goal setting, monitoring, risk perception, positive experiences, suffering, action planning, health condition, function impairments, attitude and self-efficacy. Reported environmental factors influencing dietary behaviour in wheelchair users were unadjusted kitchens, monitoring difficulties, eating out, costs, unfavourable food supply, nutrition education/counselling, access to simple healthy recipes, eating together, cooking for others, and awareness and support of family and friends.

**Conclusions:**

Important modifiable determinants of dietary behaviour in wheelchair users that might be influenced in lifestyle interventions are knowledge, fatigue, habits, self-control, intrinsic motivation, risk perception, attitude and self-efficacy. It is recommended to involve relatives, since they appear to significantly influence dietary behaviour.

## Introduction

Overweight and obesity affect approximately 58% of men and 49% of women in developed countries [1], and are even more frequent in adults with disabilities. People with mild or severe lower extremity mobility difficulties have the greatest risk of obesity, about 2.5 times greater compared to those without lower extremity mobility difficulties [2]. This increased risk is related to a decreased resting energy expenditure due to muscle atrophy and physical inactivity [3–8]. Furthermore, sitting time is associated with obesity in adults [9]. For these reasons, wheelchair users with a spinal cord injury (SCI) or lower limb amputation (LLA) can significantly benefit from a healthy lifestyle [10–12].

During the first months after a SCI, the body composition changes significantly and is characterized by a rapid decrease in muscle mass below the level of injury and an increase in fat mass [13]. Evidence shows that the body mass index (BMI) of people with SCI gradually increases during and after inpatient rehabilitation. The most pronounced increase appears in the first year after discharge from inpatient rehabilitation [14]. A high BMI is also common in persons with LLA [15,16]. Studies in persons with SCI or LLA report prevalence rates of overweight, obesity and metabolic syndrome ranging from 43 to 75 percent [14,16–20]. An increased BMI is a leading cause of preventable deaths due to the relation with cardiovascular diseases and several other (chronic) diseases [1,21]. Also, a high BMI can hinder physical functioning and independence in the short and longer term, because it leads to high loads on the upper extremities during transfers and wheelchair propulsion, and negatively affects prosthetic device use and fit [22–25]. This increases the risk of overuse injuries, upper extremity pain and increased wheelchair use [25–27]. Moreover, research has shown that a third of people with SCI experience increasing weight as a major health problem [28].

Despite this knowledge, it has been shown that persons with SCI have a poor quality diet characterized by inadequate intake of dairy, fruit, whole grain foods and fibre, and a too high intake of fat [29,30]. In persons with LLA, the intake of total fat, saturated fat, sugar and sodium have been shown to be higher than recommended [15]. Therefore, it is important that wheelchair users are encouraged to adopt a healthy diet and that healthy diet promotion interventions are targeted to this group. However, little research has focused on dietary behaviour in wheelchair users. Hall et al. examined barriers to a healthy diet in women with physical disabilities, and reported fatigue, costs of healthy foods, lack of desire or will power, shopping difficulties and lack of time to shop or prepare food as important barriers [31]. Bailey et al. explored facilitators and barriers to an anti-inflammatory diet in six persons with SCI, and identified autonomy over meal choice, experienced health benefits, social support of family and peers and use of adherence strategies as facilitators [32]. Lack of motivation, lack of knowledge, diet costs and social gatherings were the main discussed barriers in this study [32]. Littman et al. studied barriers to making dietary changes in veterans with LLA. In this group, the most reported barriers to changing dietary behaviour were habits, costs, psychological distress, lack of time, too much unhealthy food at work and home, eating out, and not knowing where to start [16].

In daily practice, Dutch rehabilitation professionals encounter many wheelchair users with SCI or LLA who have gained weight quickly after inpatient rehabilitation due to an unhealthy lifestyle. To counter this problem, they asked for the development of a digital lifestyle intervention aimed at promoting healthy diet in these target groups. As a first step in the development of this intervention, the present study aims to identify determinants of dietary behaviour in wheelchair users with SCI or LLA living in the Netherlands, from the perspectives of both wheelchair users and rehabilitation professionals. The findings should make an important contribution to the field of health promotion programs for wheelchair users.

## Methods

### Design

This study is part of the Wheelchair Exercise and Lifestyle Study (WHEELS) project, with the main objective to develop a digital intervention for wheelchair users with SCI or LLA to promote a healthy lifestyle in terms of physical activity, healthy diet and sufficient rest and relaxation. This digital lifestyle intervention in the form of a mobile application (app) will be developed for stand-alone and blended use. In the case of blended use, the lifestyle coach offers a mix of face-to-face and remote lifestyle coaching via the app. This qualitative study is part of the needs assessment to guide the development of the intervention. In this needs assessment data on determinants of dietary behaviour, physical activity and rest and relaxation were collected using focus group discussions. This paper focuses on determinants of dietary behaviour in wheelchair users. The results on determinants of physical activity were previously published in Disability and Rehabilitation [33]. The focus groups were held separately with wheelchair users and rehabilitation professionals to ensure that participants felt free to share all their thoughts. The study was approved by the Medical Ethics Committee (METC nr P1761) of the Slotervaart hospital and Reade, Amsterdam, The Netherlands.

### Participants

A purposive sample of adult wheelchair users with SCI or LLA and rehabilitation professionals (i.e. dieticians, exercise therapists, physical therapists and occupational therapists) was recruited through two exercise therapists working in local rehabilitation centres in the Netherlands: Heliomare (Wijk aan Zee) and Reade, centre for rehabilitation and rheumatology (Amsterdam). The aim of recruitment was to create heterogeneous groups, consisting of four to eight participants each, to make sure that all participants had enough time to share their thoughts and discuss the topics of interest [34–36]. Potential participants were approached face-to-face, by telephone or by email. An email with more detailed information about the study was sent to those who expressed an interest in participation.

### Setting

Six focus groups were held in June 2017. Three (two focus groups with wheelchair users and one with professionals) took place in Heliomare and three (two focus groups with wheelchair users and one with professionals) took place in Reade. A seventh focus group discussion with wheelchair users took place in October 2017 during the annual meeting day of the Dutch Spinal Cord Injury Association in Ede, The Netherlands.

### Procedures

Before each focus group, the participants signed an informed consent form and completed a short questionnaire that was used to describe the study population. For wheelchair users, this questionnaire included demographics (i.e. gender, date of birth, level of education, living situation, body height and weight, characteristics of SCI or LLA, time since SCI or amputation) and a question in which participants were asked to grade themselves on a scale of 1 (not consciously dealing) to 7 (very consciously dealing) in terms of dealing with healthy food (S1 Questionnaire). For rehabilitation professionals, this questionnaire included gender, profession, years of experience working with wheelchair users with SCI or LLA, and a 7-point scale for perceived importance of healthy food for wheelchair users (S2 Questionnaire). A semi-structured interview, guided by a moderator, was used to direct the focus group discussions. The focus group discussions took place in a conference room, lasted a maximum of 90 minutes and were audio-recorded with consent of the participants. An assistant moderator took notes to identify the respondents on the audio-recording transcription and to identify main themes. The second (L.E.A.) and third author (T.D.) fulfilled the roles of moderator and assistant moderator. They are both female (age: 26–30 years) and have experience with research among wheelchair users with multiple sclerosis (L.E.A.) and focus group moderation (T.D.). The general rules for focus group implementation were applied, i.e. each participant had the right to share one’s views and opinions, mutual respect, and no sharing of information outside the group (confidentiality) [34,37]. Furthermore, it was emphasized that all opinions were valuable and that there were no right or wrong answers. The focus groups started with an introduction to inform participants about the goal of the WHEELS-project and the focus group rules. Each participant received a reward (a power bank) following participation.

### Interview guide

Focus groups were guided by a semi-structured interview developed by the research team, and based on a format developed by Deliens et al. to identify determinants of eating behaviour in Belgian university students [38] (Table 1). The questions were carefully adjusted for the current research and extra questions were added using appropriate literature [36,37]. The research team decided to ask open questions regarding healthy lifestyle related behaviour, because we had a concurrent focus on dietary behaviour, physical activity and relaxation behaviour. After all, the focus groups were part of a needs assessment for a lifestyle intervention focused on these three pillars. The questions were not specified towards dietary behaviour to ensure that all participants could express what they considered most important regarding lifestyle. When the participants did not discuss this pillar in response to the questions, they were additionally asked to answer the questions specifically for dietary behaviour.

**Table 1:** Interview guide for the focus groups.

Question type	Questions wheelchair users	Questions professionals
**Opening**	Can you introduce yourself?	Can you introduce yourself?
**Problem**	What do you do to stay healthy?	Do wheelchair users in general have a healthy lifestyle?
	Are you satisfied with your current lifestyle?	Do you feel that most wheelchair users are aware of the importance of a healthy lifestyle?
	Which barriers and enablers do you encounter in working on a healthy lifestyle?	What are the most important lifestyle problems in wheelchair users after inpatient rehabilitation?
**Key**	What needs to change to improve your current lifestyle?	Which factors prevent the development of a healthy lifestyle in wheelchair users?
	What do you need to adopt a healthy lifestyle?	What can be improved for wheelchair users to make it easier to adopt a healthy lifestyle?
	What keeps you motivated to maintain a healthy lifestyle?	What do wheelchair users need to maintain a healthy lifestyle?
**Ending**	Do you have any questions, remarks, suggestions or additions?	Do you have any questions, remarks, suggestions or additions?

### Data analysis

**Fig 1:**
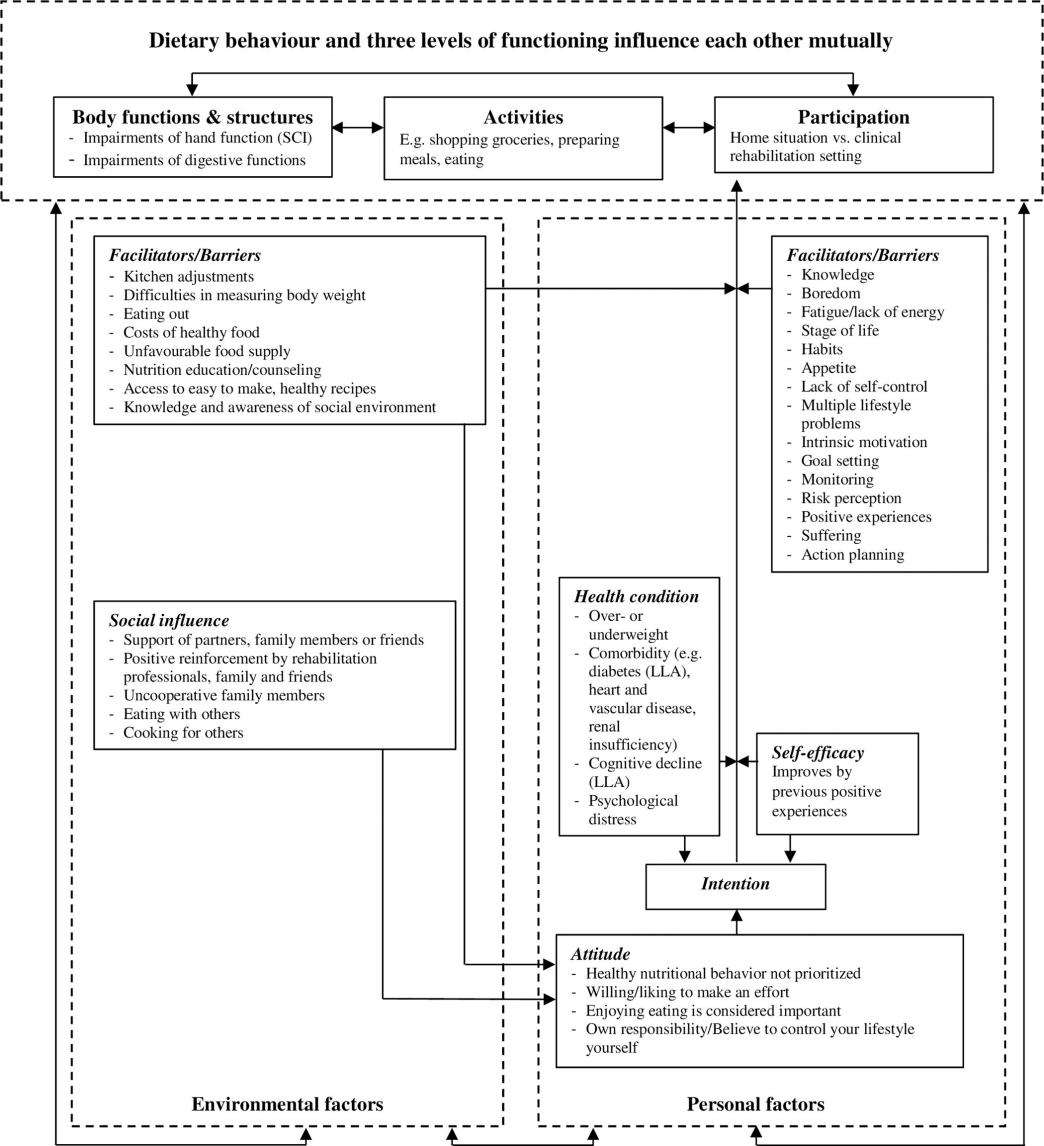
Summary of the focus group results displayed in the slightly adapted* Physical Activity for people with a Disability (PAD) model*. Slightly adapted means that “physical activity” is replaced with “dietary behaviour”. The determinants assigned to each category are presented in random order. LLA = lower limb amputation; SCI = spinal cord injury.

After the focus groups, the moderator and assistant-moderator evaluated and discussed the most notable emotions, statements and themes. The audio recordings were transcribed verbatim, after which they were analysed using a thematic content analysis approach [39]. Three types of coding were used: open, axial and selective coding [40]. The focus groups of the wheelchair users and the professionals were coded separately. Initial codes were created by coding text segments (open coding). The open codes were then compared and subcategorized in superordinate codes (axial coding). To classify areas of determinants related to healthy nutrition behaviour (selective coding), we used the Physical Activity for people with a Disability (PAD) model as a theoretical framework [41]. This model is a combination of the International Classification of Functioning, Disability and Health (ICF) model and the Attitude, Social influence and self-Efficacy (ASE) model [42,43]. The PAD-model is developed as a theoretical framework for interventions and research on physical activity promotion for people with a disability. Because physical activity and dietary behaviour are both lifestyle behaviours that impact general health and well-being, and both the ICF-model and ASE-model are used in nutrition research, the research team considered the model suitable as a framework for the present study. The model was slightly adapted, by replacing the term “physical activity” with the term “dietary behaviour”. According to the PAD-model, the determinants were coded as personal or environmental determinants of dietary behaviour, whereby personal determinants were further categorized into facilitator, barrier, health condition, attitude, intention, and self-efficacy, and environmental determinants were further categorized into facilitator, barrier and social influence (Fig 1). The analyses were carried out by two researchers (J.F.M.H. and D.H.) to ensure reliability of coding and data interpretation. Disagreements were discussed and in case of persisting disagreements, a third researcher was consulted (M.D.). The transcripts were analysed with MAXQDA version 11 (VERBI GmbH, Berlin, Germany). The data obtained from the questionnaire were analysed with SPSS Statistics version 24 (IBM, Armonk, United States). Self-reported body height and weight were used to classify the wheelchair users in BMI categories. For participants with LLA the BMI was calculated based on the post-amputation adjusted body weight [44,45]. To account for the missing limb(s), the body segment proportions presented by Durkin & Dowling [46] and Rosenberg [25], and the method proposed by Osterkamp [44] and Himes [45] were used. A post-amputation adjusted BMI of <25 was considered normal weight, a BMI of 25–29.9 was considered overweight, and a BMI of ≥30 was considered obese [47]. For participants with SCI adjusted BMI categories were used based on Gater [48] and Laughton et al. [49], whereby a BMI of <22 was considered normal weight, a BMI of 22–24.9 was considered overweight and a BMI of ≥25 was considered obese.

## Results

### Participants

A total of 25 wheelchair users participated in five focus group discussions. In two additional focus groups, 11 rehabilitation professionals were involved. Sample characteristics, obtained with a self-report questionnaire prior to the focus groups, of the wheelchair users and professionals are described in Table 2.

**Table 2:** Characteristics of the wheelchair users (n = 25) and professionals (n = 11) who participated in the focus groups.

**Wheelchair users (n = 25)**	
Age in years, median (range)	58 (39–75)
Male/female, n	13/12
Educational level, n	
Primary school/secondary school/higher professional education or university	1/10/14
Spinal cord injury/amputation, n	17/8
Spinal cord injury, n	
Paraplegia/tetraplegia/unknown	12/3/2
Complete/incomplete lesion/unknown	9/6/2
Amputation, n	
Single leg above the knee	4
Single leg below the knee	2
Double leg above the knee	1
Double leg at the knee (knee disarticulation)	1
Self-reported cause^a^: trauma/diabetes/infection/congenital disorder	2/3/2/1
Time since (first) spinal cord injury or lower limb amputation in years, median (range)	9 (0–55)
Living situation, n	
Home/inpatient rehabilitation	23/2
Body mass index category^b^, n	
Normal weight/overweight/obese/unknown	3/7/12/3
This grade (1 not consciously dealing; 7 very consciously dealing) applies to me for consciously dealing with healthy food, median (range):	5.5 (3–7)
**Professionals (n = 11)**	
Male/female, n	1/10
Profession, n	
Physical therapist	3
Exercise therapist	3
Exercise therapist and research assistant	1
Dietician	2
Occupational therapist	1
Occupational therapist and researcher rehabilitation medicine	1
Years of working experience with wheelchair users, median (range)	10 (1–30)
This grade (1 unimportant; 7 very important) is appropriate for the importance of healthy food for wheelchair users, median (range):	7 (6–7)

^a^ The causes of amputation reported here are based on oral self-report during the focus-group discussions.

^b^ For participants with SCI the adjusted BMI categories for people with SCI were used (normal weight: BMI <22 kg/m^2^; overweight: 22 ≤ 25; obese: BMI ≥25). The BMI for wheelchair users with lower limb amputation was adjusted for missing limb(s), after which the BMI categories for the general population were used (normal weight: BMI <25 kg/m^2^; overweight: 25 ≤ 30; obese: BMI ≥30). References for these estimations, methods and classifications are described in the methods section.

### Themes

The axial codes that emerged from the thematic content analysis conveniently fitted into the slightly adapted PAD-model (Fig 1). Therefore, the components of this model were used as themes to categorize the results (selective codes). These themes were:

- body functions & structures;
- personal factors, with the sub-themes of barriers, facilitators, health condition;
- attitude and self-efficacy; and
- environmental factors, with the sub-themes of barriers, facilitators and social influence.

The results of the focus groups with wheelchair users and focus groups with professionals are presented together and are ordered using the aforementioned themes. An overview of which determinant was discussed in which group, i.e. wheelchair users and/or professionals, is provided in a supplementary table (S1 Table).

### Body functions & structures

Professionals mentioned *impairments of hand function* to affect healthy cooking behaviour of wheelchair users with SCI. Wheelchair users, both with SCI and LLA, discussed the negative influence of *impairments of digestive functions*, resulting for example in the use of tube feeding, on their dietary behaviour.

“*People around you talk about food*. *That is always*. *About the weather and about food*. *You notice this when you cannot eat*. *From misery you go away at dinner time*. *Then you sit in a corner with your face to the wall*. *That is very heavy*. *To become hysterical*.*”* (Focus group 5, female, age: 39 years, single leg amputation below the knee for 1 year)

*“I agree*, *I experienced that too*. *I could hardly eat at the beginning of rehabilitation*. *I have learned to eat ground food at a given time*.*”* (Focus group 5, male, age: 39 years, incomplete SCI for 3 years)

### Personal factors

#### Barriers

Several personal barriers to healthy dietary behaviour were discussed by both wheelchair users and professionals. The barrier of healthy dietary behaviour that was mentioned most frequently by wheelchair users and professionals was *knowledge*: many wheelchair users do not know to what extent their energy- and protein requirements have changed due to their injury or amputation. This makes it difficult to estimate how much they should ideally eat. Some wheelchair users also indicated not to know what healthy food is.

A second barrier reported by professionals was *lack of intrinsic motivation*. When wheelchair users are not motivated it is very difficult to change their dietary behaviour.

“*People who do not really want*, *or do not see the importance*, *cannot be persuaded*, *not even with such an app [lifestyle app]* …… *You must be somewhat motivated*.*”* (Focus group 3, professional, 3 years of working experience with wheelchair users)

A third barrier reported by both professionals and wheelchair users was being bored and having lots of time to eat (*boredom*).

*“You do gain weight*, *but that is partly due to boredom*. *I worked before but have now been declared unfit for work*. *If you are at home all day*, *you get something to eat and drink more easily than you would do at work*.*”* (Focus group 2, male, age: 54 years, single leg amputation below the knee for 10 years)

A fourth barrier discussed by both groups was that doing groceries and cooking healthy recipes are time- and energy-consuming activities for wheelchair users. In particular *fatigue* or *lack of energy* was often referred to as a barrier to healthy dietary behaviour.

“*I also notice this*. *That I have too little energy to go through a recipe completely*. *Then there are again several products that I do not know where to buy*. *Buying something that is not around the corner is a real hassle for me*.*”* (Focus group 1, male, age: 62 years, complete SCI for 2 years)

Fifth, both groups believed that the *stage of life* a wheelchair user is in influences their lifestyle. Shortly after the clinical rehabilitation phase, the minds of people with a recent SCI or LLA are not yet ready to engage in a healthy lifestyle. They have other priorities. After a while, when they have got their lives back together, they are more open for working on a healthy lifestyle. Another example of the influence of stage of life is that wheelchair users with young children mentioned that caring for their children leads to less time to work on a healthy lifestyle.

A sixth and seventh barrier expressed by both wheelchair users and professionals were eating *habits* learnt at home and lack of *appetite*. One wheelchair user mentioned appetite not as a barrier but as a facilitator of healthy dietary behaviour. Because he found wine less tasty since his injury, he started to drink less alcohol.

In addition, wheelchair users revealed that lack of *self-control* (e.g. emotions triggering the urge to eat comfort food) negatively influenced their dietary behaviour.

Finally, professionals felt that engaging in *multiple unhealthy lifestyle behaviours* reduces the chance of health behaviour change, because changing in different aspects requires extra commitment.

#### Facilitators

A facilitator for healthy dietary behaviour that was emphasized by professionals, and was also discussed by wheelchair users, was willpower or *intrinsic motivation*.

“*No*, *all those stimuli from outside*, *so all that good advice and stuff*, *that's all nice*, *but it has to come from yourself*.*"* (Focus group 2, male, age: 71 years, complete SCI for 3 years)

In addition, both groups believed that *goal setting* and *monitoring* of energy intake and weight facilitates making healthy food choices.

“*Here [rehabilitation centre] is a scale that I can use*. *Getting on it made me see where I stood for the first time*. *It motivated me to lose weight*.*”* (Focus group 1, male, age: 53 years, complete SCI for 46 years)

Regarding goal setting, the professionals indicated the importance of setting small feasible sub goals.

Other facilitators of healthy nutritional behaviour discussed by both groups were *risk perception* and having previous *positive experiences* with healthy eating, leading to enhanced self-efficacy and motivation to maintain the behavioural change. Finally, the professionals mentioned *suffering* from the negative consequences of unhealthy dietary behaviour and having an *action plan* as facilitators for change towards a healthy diet.

#### Health condition

According to both groups *under- and overweight* may affect the intention and need to change dietary behaviour. In addition, in most focus groups the influence of *comorbidity* on dietary behaviour was discussed. Two wheelchair users with LLA told that they had diabetes mellitus which forced them to pay attention to their dietary pattern. One wheelchair user with SCI told that he started eating less salt and phosphate-rich foods due to renal insufficiency. Professionals stated that heart and vascular disease can influence dietary behaviour through risk perception. Wheelchair users with these conditions are often more aware that they can reduce their risk of worsening of disease by eating healthier. Finally, professionals discussed *cognitive decline* (in wheelchair users with LLA) and *psychological distress* as factors negatively influencing dietary behaviour. Cognitive decline was assumed to reduce the initiative to change behaviour, and psychological distress was mentioned in relation to emotional eating.

“*If you feel sad*, *you will start to search for food earlier*. *Then emotional eating comes up*. *I do really think that this plays a role*.*”* (Focus group 6, professional, 10 years of working experience with wheelchair users)

#### Attitude

Both wheelchair users and professionals believed that the *attitude* of wheelchair users regarding their dietary behaviour is an important determinant. Many wheelchair users do not prioritize healthy eating enough. This was mentioned in both groups but particularly emphasized by the professionals. At the same time, most wheelchair users indicated that they really wanted to eat healthier and were willing to put more effort into this.

“*So I want to try to shift all the attention that now goes to those dirty cigarettes to becoming enthusiastic about good cooking and healthy recipes*. *That is the goal*. *Whether it will work…*.*”* (Focus group 1, female, age: 44 years, complete SCI for 8 years)

Factors that were mentioned to hinder wheelchair users in healthy eating were that doing groceries, searching for healthy recipes and cooking are perceived as physically demanding and not fun. Furthermore, wheelchair users indicated that healthy eating should not be at the expense of being able to enjoy food and cosiness or socializing at the table. Attention was also paid to responsibility. Both groups felt that wheelchair users are responsible for their lifestyle themselves and that if you want to change your dietary behaviour, it is important to believe that you have control over it.

*“Speaking for myself*, *I know that I have to blame myself*. *That I am less healthy is my own fault*.*”* (Focus group 2, male, age: 54 years, single leg amputation below the knee for 10 years)

#### Self-efficacy

The focus group discussions with professionals showed that they consider *self-efficacy* as an important determinant of dietary behaviour. They explained that previous positive experiences with a healthy diet lead to an increase in self-efficacy, which helps to improve dietary behaviour again.

“*It starts with a success experience*. *Hell*, *I can do this*! *Now let me see what is next*.*”* (Focus group 6, professional, 15 years of working experience with wheelchair users)

### Environmental factors

#### Barriers

Wheelchair users discussed multiple barriers in their environment that complicate healthy dietary behaviour. Often, *kitchens are not appropriately adjusted* to the wheelchair user’s needs. A frequent problem is that the kitchen counter, stove and sink are too high. This leads to overuse of their shoulders. Furthermore, lifting heavy cooking pans can be dangerous.

“*I recently have an adapted kitchen with knee space below the gas stove*. *But actually*, *you sit too high*. *I have to cut like this [bends forward] or I have to cut with the chopping board on my lap*. *That is how I do it*. *However*, *taking a heavy pan from the fire which is so high*, *is actually life-threatening*.*”* (Focus group 1, male, age: 62 years, complete SCI for 2 years)

*“In the past*, *this was not allowed*: *if you got an adapted kitchen from the municipality you could not have a gas stove*, *and rightly so*. *I once set my sleeve on fire*. *It is life-threatening*.*”* (Focus group 1, female, age: 58 years, double leg amputation above the knee for 55 years)

A second environmental barrier of healthy dietary behaviour mentioned by wheelchair users was *difficulty with weighing oneself*. Most wheelchair users do not have an accessible scale in their environment, which makes it difficult to monitor weight. Being unaware of weight and weight fluctuations does not stimulate dietary changes.

“*I have*, *since my 35*^*th*^ *or so*, *problems with my weight*. *I used to exercise a lot until I started my own company and suddenly stopped*. *Then I became much heavier*. *I did not know that*. *I cannot just stand on a scale*, *and when I sit on it*, *I cannot see how heavy I am*. *Years went by before I was weighed……… It appeared—what I really thought was terrible—that I weighed 92 kilos*.*”* (Focus group 1, age: 53 years, complete SCI for 46 years)

Third wheelchair users believed that *eating out* hampers healthy dietary behaviour. Due to boredom and dreading doing groceries and cooking, some wheelchair users tend to eat out more often. In a restaurant you are less inclined to eat and drink healthy as at home. As one wheelchair user said: you always eat a bit more than at home and take more often a dessert.

Fourth, wheelchair users mentioned the *costs* of healthy food as a barrier to healthy dietary behaviour.

*“Well*, *what I still find a barrier are the costs*. *I would also like to do some healthier shopping and purchase healthier products*, *but that is often very expensive*.*”* (Focus group 4, female, age: 47 years, single leg amputation above the knee for 31 years)

Finally, wheelchair users addressed the food supply during inpatient rehabilitation which lacked variety and good taste *(unfavourable food supply)*. This had not helped them in developing healthy dietary behaviour.

“*The food here [rehabilitation centre] is nothing to write home about* … *It is just inedible*. *But then you have to eat because otherwise you just cannot keep it up*. *So you also eat unhealthy things*.*”* (Focus group 4, male, age: 54 years, single leg amputation above the knee for 5 months)

#### Facilitators

Both professionals and wheelchair users emphasized that *health and nutrition education and counsellin*g during and after the rehabilitation period is an important facilitator of healthy dietary behaviour. Education is needed to make wheelchair users aware of the changed energy and nutrient needs, due to their injury and possible secondary consequences such as decubitus. Both groups noticed that they felt it advisable to start this education and counselling during inpatient rehabilitation and to continue in the home situation.

“*You are pampered in the rehabilitation centre*, *and then the moment you step outside*, *you fall into a huge deep black hole*. *What I really missed is a gradual reduction in contact*, *from once every six months to once a year*. *That they ask you to come back and how you are doing*. *Like*: *“I see that you have gained 20 kg*, *we have to do something about your diet*.*” You miss general guidance when you leave the rehabilitation centre*.*”* (Focus group 2, male, age: 59 years, incomplete SCI for 5 years)

In addition, wheelchair users suggested that *access to easy to make*, *healthy recipes* facilitates healthy dietary behaviour.

“*Eating is a very difficult topic for me*, *because I like to eat but I hate to cook*. *But I hope to change that*. *What can help me are recipes*.*”* (Focus group 1, female, age: 44 years, complete SCI for 8 years)

Finally, *knowledge and awareness of the social environment* was mentioned by both wheelchair users and professionals as a prerequisite for being able to support wheelchair users in adopting a healthy lifestyle.

“*I have a wife at home who corrects me when she thinks that I am talking nonsense*. *And uhm… yes*, *she does take that [lower energy requirements] into account*. *So*, *I have a sort of sparring partner …”* (Focus group 2, male, age: 71 years, complete SCI for 3 years)

“*Yet your wife is not knowledgeable about spinal cord injury*, *she does not know much about an altered metabolism*.*”* (Focus group 2, male, age: 59 years, incomplete SCI for 5 years)

“*My wife immediately read a book*, *so she knows everything now*. *But without a joke*, *you have to dig into it a bit*, *and if you do that yourself and your partner does that*, *or your housemates do that*, *that makes a difference*.*”* (Focus group 2, male, age: 71 years, complete SCI for 3 years)

“*I would like to emphasize that*, *in terms of lifestyle*, *you cannot manage it [change] if you only approach the individual*. *Certainly with regard to nutrition*, *we can come up with a number of examples in which the system also maintains something*.*”* (Focus group 6, professional, 10 years of working experience with wheelchair users)

#### Social influence

The role of the social environment was found to be twofold. On the one hand, *support of partners or family members* and *positive reinforcement by rehabilitation professionals*, *family and friends* were mentioned as factors helping wheelchair users to eat healthy.

“*I appreciate external motivation*. *When the dietician says*, *“You have lost some weight again”*, *and I see results at the end of my diet*, *then I am very happy*.*”* (Focus group 2, male, age: 59 years, incomplete SCI for 5 years)

“*I also experience that when you get confirmation in what you are doing*, *that people see it and say*, *“well done”*, *that certainly gives an incentive to continue*. *…”* (Focus group 2, female, age: 58 years, complete SCI for 22 years)

Most wheelchair users indicated that it is not possible to make healthy food choices alone. This requires help, understanding and cooperation from partners and housemates. The rehabilitation professionals underlined the significance of involving family members in education on healthy eating.

On the other hand, wheelchair users and professionals mentioned that partners and family do not always cooperate in the development and maintenance of a healthy diet [*uncooperative family members*]. Often, they are not aware of the reduced energy consumption and the importance of healthy eating, now that their partner or parent leads a sedentary life. For example, one wheelchair user told that she sometimes did dinner competitions with her son aimed at eating the largest portion.

In addition, wheelchair users indicated that *cooking for others* and *eating together* encourages more extensive and healthier cooking.

“*Cooking for myself is not easy*. *I do can cook*. *I used to cook for my wife*. *She has been ill for a long time*. *But that is quite tiring from your wheelchair*. *And for myself*. *I do not get any further than a salad*. *That is why I often invite people to eat together*. *They then cook more extensively*. *Personally*, *I find that difficult*.*”* (Focus group 1, male, age 62 years, complete SCI for 2 years)

## Discussion

As part of a needs assessment to guide the development of a digital intervention aimed at promoting a healthy lifestyle in wheelchair users with SCI or LLA, the objective of the present focus group study was to gain insight into determinants of dietary behaviour. Several personal determinants were identified, of which some are believed to be modifiable and may be targeted in the intervention. For example, consideration should be given to provide information about healthy food, energy and protein requirements. To prevent fatigue from hindering an active lifestyle, guidance could be included on distributing the available energy throughout the day. In addition, it seems useful to teach skills to break habits and to resist unhealthy temptations, such as action and coping planning [50]. The participants also discussed unmodifiable personal determinants that can be used to tailor the intervention to subgroups, for example stage of life, physical impairments and comorbidity. In addition to nutrition education and counselling by professionals, knowledge, awareness and support from partners, family and friends were mentioned as important modifiable environmental factors influencing dietary behaviour. It seems therefore important to also involve the social environment in the intervention.

In line with the studies of Hall et al. [31], Bailey et al. [32] and Littman et al. [16] in respectively women with physical disabilities, persons with SCI and veterans with LLA, our study population mentioned fatigue, lack of intrinsic motivation, lack of knowledge, lack of time, habits, costs of healthy foods, unhealthy food supply and eating out as important personal and environmental barriers to healthy dietary behaviour. However, in the study of Bailey et al. [32], “lack of knowledge” related to meal planning and preparation to adhere to an anti-inflammatory diet, while in our study this related to what healthy food is and energy and protein requirements. In our study “lack of time” was subcategorized into “not prioritizing healthy eating” and placed in the subtheme “attitude”. It was notable that both “lack of time” and “having lots of time” were mentioned as a barrier of healthy dietary behaviour by wheelchair users. Participants with a busy, active life felt that they had too little time to cook healthily, and participants with a less active life, for example because they had lost their job, indicated that they were inclined to eat too much out of boredom.

Perceived facilitators of healthy dietary behaviour were, in contrast, less studied by Hall et al. [31] and Littman et al. [16] or by others. Bailey et al. [32] explored facilitators of adherence to an anti-inflammatory diet, and in accordance with us, identified social support and having positive experiences, e.g. in the form of weight loss, as facilitators. For us it was important to ask what wheelchair users need to adopt a healthy lifestyle and stay motivated, because this study was part of a needs assessment for the development of a digital lifestyle intervention for wheelchair users. When comparing the results of the focus groups with wheelchair users and professionals, it is noticeable that wheelchair users discussed more environmental barriers, while professionals mainly discussed personal barriers that wheelchair users can often change themselves. In addition to the role of the social environment, environmental barriers were hardly discussed by the professionals. Because the professionals evidently had knowledge of behavioural change principles, they discussed the needs of wheelchair users (personal facilitators) in more detail. An example of this is that both groups indicated goal setting as a useful method for eliciting behaviour change. Professionals went more in depth and suggested that goals should be feasible and broken down into smaller steps. Both wheelchair users and professionals mentioned nutrition education and counselling as environmental facilitators of healthy dietary behaviour. Regarding nutrition education and counselling, some wheelchair users indicated that this was missing in their rehabilitation programme. This can be explained by the fact that they completed rehabilitation years ago. In the past the dietician in the rehabilitation centres in the Netherlands mainly treated undernutrition. Nowadays there is much more attention for the prevention of overweight and healthy dietary habits. Furthermore, the participants mentioned several self-regulatory factors, such as self-control, prioritizing and self-efficacy, and self-regulation methods, such as goal setting and monitoring of energy intake and weight, as factors facilitating healthy eating behaviour. Based on these results, motivational interviewing, a technique that targets self-regulation, might be a powerful method to guide wheelchair users to healthy dietary behaviour. Motivational interviewing is a client-oriented counselling technique that helps people to investigate ambivalent feelings, develop intrinsic motivation, decide what is needed to change and deal with difficulties during the process [51]. The starting point of motivational interviewing is that the motivation to change comes from within the individual and is not imposed from outside. Both wheelchair users and professionals confirmed this starting point by mentioning intrinsic motivation as a key determinant of healthy dietary behaviour. Motivational interviewing has already been successfully used in lifestyle interventions aimed at increasing physical activity and reducing weight in people with SCI or LLA [52,53]. Motivational interviewing can be used well in blended use of the mobile lifestyle app to be developed.

A strength of the current study is that it combines the insights of both rehabilitation professionals and wheelchair users. Where wheelchair users approach dietary behaviour from their own perspective, rehabilitation professionals place the topic in a broader context, and link the dietary problems they observe to their known intervention strategies. By focusing the digital lifestyle intervention to be developed on determinants that are considered important by both wheelchair users and rehabilitation professionals, it is more likely that the intervention will target factors that do affect dietary behaviour of wheelchair users and will be offered by rehabilitation professionals. After all, the intended target group and providers of an intervention are best able to interpret the perspectives and needs of the groups to which they belong [51].

Some comments must be made about the applicability of the present study’s results in practice. It is important to keep in mind that the focus groups with the wheelchair users and rehabilitation professionals were performed separately. This method was chosen to ensure that each participant felt free to talk openly and to contribute to the discussion. However, this method prevented interaction between the two groups from which additional results could have emerged. Furthermore, selection bias may have occurred. Participants who were already interested in a healthy lifestyle were more inclined to participate in group discussions on this topic. This is supported by the high grade the wheelchair users gave themselves for engagement in healthy food behaviour (Table 1). However, according to the adjusted BMI calculations for wheelchair users with SCI or LLA [25,44–49], respectively 19 of the 25 wheelchair users were overweight or obese. This indicates that most participating wheelchair users did not have a healthy weight despite their self-reported engagement in healthy food behaviour. Because these BMI figures are based on self-report, they must be interpreted with caution. Apart from the general interest in a healthy lifestyle, the groups were quite heterogeneous. The wheelchair users differed in level of SCI or amputation, time since injury, cause of amputation, age, gender and educational level. So, there were several potential user groups of the digital lifestyle intervention represented in this study. The group of participating rehabilitation professionals was also diverse. They varied for example in profession and years of experience with wheelchair users. Focus groups are ideally held in heterogeneous groups because this stimulates the discussion. A disadvantage of the variation in professions, however, was that not all professionals were equally specialized in dietary behaviour. There were only two dieticians, divided over the two focus groups, and no health psychologists included. Because, in the Netherlands, rehabilitation professionals pay attention to behavioural change when coaching patients in self-management, and the participating professionals were highly interested in lifestyle change, we are confident that they had sufficient knowledge of the dietary behaviour of wheelchair users to discuss determinants thereof. Nevertheless, inclusion of more dieticians and health psychologists may have yielded additional specialized knowledge about the dietary behaviour of wheelchair users. Finally, we did not let the participants check the transcripts for accuracy due to ethical and feasibility considerations. In addition, experience shows that participants make little use of the possibility of checking transcripts [40]. This is a limitation of our study because member checking could have improved the trustworthiness of our results.

This study provides insights on dietary behaviour that are valuable for the development of a digital lifestyle intervention for wheelchair users with SCI or LLA. Lifestyle interventions that target dietary behaviour are of great importance since wheelchair users with SCI or LLA often die from the effects of cardiovascular and/or metabolic-endocrine disorders [54–56]. Major risk factors for these disorders are obesity and insulin resistance, which are largely attributed to poor dietary behaviour [54]. Determinants of dietary behaviour that could be targeted in future lifestyle interventions for wheelchair users with SCI or LLA are knowledge of healthy food, energy and protein requirements (which food products to buy and how much of the products to eat), fatigue, habits, self-control, intrinsic motivation, risk perception, attitude and self-efficacy. Determinants that cannot be changed but are also relevant according to wheelchair users and rehabilitation professionals are stage of life, impairments of hand function, impairments of digestive functions and comorbidity. These determinants could be used to personalize the intervention. In addition, it is important to involve the social environment in the intervention, because knowledge, awareness and support from partners, family and friends were also identified as influential factors affecting dietary behaviour.

## Supporting information

S1 Questionnaire Short questionnaire completed by the wheelchair users prior to the focus group. (DOCX)

S2 Questionnaire Short questionnaire completed by the rehabilitation professionals prior to the focus group. (DOCX)

S1 Table Overview of the determinants of dietary behaviour in wheelchair users with SCI or LLA discussed by wheelchair users and rehabilitation professionals. (DOCX)
