# Supplementary material for: Determinants of dietary behaviour in wheelchair users with spinal cord injury or lower limb amputation: Perspectives of rehabilitation professionals and wheelchair users
Source: PLoS One. 2020 Jan 31;15(1):e0228465. doi: 10.1371/journal.pone.0228465 (PMC6993975; doi:10.1371/journal.pone.0228465)
Supplement: S1 Questionnaire — (DOCX) [file pone.0228465.s001.docx]

**S1 Questionnaire. Short questionnaire completed by the wheelchair users prior to the focus group.**

**Original version (Dutch)**

**Vragenlijst deelnemers focusgroep WHEELS-project**

Focusgroep nr.: ____ Datum: ___/___/______

Deelnemer nr.: ____

Geslacht: ⃝ Man ⃝ Vrouw

Geboortedatum: ___/___/_______ D/M/J

Lengte in cm: _______________

Gewicht in kg: _______________

***Invullen wat van toepassing is:***

**Dwarslaesie:**

Niveau dwarslaesie: _________

⃝ Volledige laesie ⃝ Partiële laesie

Dwarslaesie sinds: ______ Jaar

**Amputatie**

Niveau amputatie:

O Bekkenamputatie

O Heupamputatie

O Bovenbeenamputatie

O Knieamputatie

O Onderbeenamputatie

O Enkelamputatie

O Dubbelzijdige amputatie, nl:

links: _________________________

rechts: _________________________

Amputatie sinds: ______ jaar

Wat is uw hoogst afgeronde opleiding?

O Geen opleiding

O lagere school / basisonderwijs

O LBO, VBO, LTS, LHNO, VMBO, MULO

O MAVO, VMBO-t, MBO-kort

O MBO, MTS, MEAO

O HAVO, VWO, Gymnasium

O HBO, HEAO, PABO, HTS

O Universiteit

O Anders, namelijk: _________________________

Wat is uw huidige woonsituatie? ⃝ Revalidatiecentrum ⃝ Thuis

Sinds: ___/___/_______ D/M/J

Wat is uw huidige werksituatie?

O Fulltime

O Parttime

O Geen werk

O Arbeidsongeschikt verklaard: ___%

O Studie

O Vrijwilligerswerk

Dit cijfer geef ik mijzelf voor het bewust omgaan met voldoende beweging:

| **Niet bewust mee bezig** |  |  | **Neutraal** |  |  | **Heel bewust mee bezig** |
| --- | --- | --- | --- | --- | --- | --- |
| 1 | 2 | 3 | 4 | 5 | 6 | 7 |

Dit cijfer geef ik mijzelf voor het bewust omgaan met gezonde voeding:

| **Niet bewust mee bezig** |  |  | **Neutraal** |  |  | **Heel bewust mee bezig** |
| --- | --- | --- | --- | --- | --- | --- |
| 1 | 2 | 3 | 4 | 5 | 6 | 7 |

Dit cijfer geef ik mijzelf voor het bewust omgaan met de balans tussen inspanning en ontspanning:

| **Niet bewust mee bezig** |  |  | **Neutraal** |  |  | **Heel bewust mee bezig** |
| --- | --- | --- | --- | --- | --- | --- |
| 1 | 2 | 3 | 4 | 5 | 6 | 7 |

**Translated version (English)**

**Questionnaire participants focus group WHEELS-project**

Focus group nr.: ____ Date: ___/___/______

Participant nr.: ____

Gender: ⃝ Male ⃝ Female

Date of birth: ___/___/_______ D/M/Y

Height in cm: _______________

Weight in kg: _______________

***Fill in what applies to you:***

**Spinal cord injury**

Level of spinal cord injury: _________

⃝ Complete spinal cord injury ⃝ Incomplete spinal cord injury

Spinal cord injury since: ______ years

**Amputation**

Level of amputation:

O Pelvic amputation

O Hip amputation

O Thigh amputation

O Knee amputation

O Lower leg amputation

O Ankle amputation

O Bilateral amputation, namely:

left: _________________________

right: _________________________

Amputation since: ______ years

What is your highest level of education?

O No education

O Primary school / primary education

O Lower vocational education, pre-vocational secondary education (LBO, VBO, LTS, LHNO, VMBO, MULO)

O General secondary education / short secondary vocational education (MAVO, VMBO-t, MBO-kort)

O Secondary vocational education (MBO, MTS, MEAO)

O Senior general secondary education (HAVO) / pre university education (VWO)

O Higher professional education (HBO, HEAO, PABO, HTS)

O University education (WO)

O Other, namely: _________________________

What is your current living situation? ⃝ Rehabilitation centre ⃝ Home

Since: ___/___/_______ D/M/Y

What is your current work situation?

O Full-time

O Part-time

O No work

O Declared incapacitated for work: ___%

O Study

O Volunteer work

This grade applies to me for conscious engagement in sufficient physical activity:

| **Not consciously engaged** |  |  | **Neutral** |  |  | **Very consciously engaged** |
| --- | --- | --- | --- | --- | --- | --- |
| 1 | 2 | 3 | 4 | 5 | 6 | 7 |

This grade applies to me for consciously dealing with healthy food:

| **Not consciously dealin** |  |  | **Neutral** |  |  | **Very consciously dealing** |
| --- | --- | --- | --- | --- | --- | --- |
| 1 | 2 | 3 | 4 | 5 | 6 | 7 |

This grade applies to me for conscious engagement in keeping a good balance between activity and rest:

| **Not consciously engaged** |  |  | **Neutral** |  |  | **Very consciously engaged** |
| --- | --- | --- | --- | --- | --- | --- |
| 1 | 2 | 3 | 4 | 5 | 6 | 7 |
