# Supplementary material for: Determinants of dietary behaviour in wheelchair users with spinal cord injury or lower limb amputation: Perspectives of rehabilitation professionals and wheelchair users
Source: PLoS One. 2020 Jan 31;15(1):e0228465. doi: 10.1371/journal.pone.0228465 (PMC6993975; doi:10.1371/journal.pone.0228465)
Supplement: S2 Questionnaire — (DOCX) [file pone.0228465.s002.docx]

**S2 Questionnaire. Short questionnaire completed by the rehabilitation professionals prior to the focus group.**

**Original version (Dutch)**

**Vragenlijst deelnemers focusgroep WHEELS-project**

Focusgroep nr.: ____ Datum: ___/___/______

Deelnemer nr.: ____

Geslacht: ⃝ Man ⃝ Vrouw

Werkzaam bij Reade/Heliomare als: ____________________________________

Aantal jaar ervaring met rolstoelgebruikers: ____________________________________

Dit cijfer geef ik voor het belang van voldoende beweging bij rolstoelgebruikers:

| **Onbelangrijk** |  |  | **Neutraal** |  |  | **Zeer belangrijk** |
| --- | --- | --- | --- | --- | --- | --- |
| 1 | 2 | 3 | 4 | 5 | 6 | 7 |

Dit cijfer geef ik voor het belang van gezonde voeding bij rolstoelgebruikers:

| **Onbelangrijk** |  |  | **Neutraal** |  |  | **Zeer belangrijk** |
| --- | --- | --- | --- | --- | --- | --- |
| 1 | 2 | 3 | 4 | 5 | 6 | 7 |

Dit cijfer geef ik voor het belang van het vinden van een goede balans tussen inspanning en ontspanning bij rolstoelgebruikers:

| **Onbelangrijk** |  |  | **Neutraal** |  |  | **Zeer belangrijk** |
| --- | --- | --- | --- | --- | --- | --- |
| 1 | 2 | 3 | 4 | 5 | 6 | 7 |

**Translated version (English)**

**Questionnaire participants focus group WHEELS-project**

Focus group nr.: ____ Date: ___/___/______

Participant nr.: ____

Gender: ⃝ Male ⃝ Female

Working at Reade/Heliomare as: ____________________________________

Number of years of experience with wheelchair users: ____________________________________

This grade is appropriate for the importance of sufficient physical activity in wheelchair users:

| **Unimportant** |  |  | **Neutral** |  |  | **Very important** |
| --- | --- | --- | --- | --- | --- | --- |
| 1 | 2 | 3 | 4 | 5 | 6 | 7 |

This grade is appropriate for the importance of healthy food for wheelchair users:

| **Unimportant** |  |  | **Neutral** |  |  | **Very important** |
| --- | --- | --- | --- | --- | --- | --- |
| 1 | 2 | 3 | 4 | 5 | 6 | 7 |

This grade is appropriate for the importance of a good balance between activity and rest in wheelchair users:

| **Unimportant** |  |  | **Neutral** |  |  | **Very important** |
| --- | --- | --- | --- | --- | --- | --- |
| 1 | 2 | 3 | 4 | 5 | 6 | 7 |
