# Supplementary material for: Determinants of dietary behaviour in wheelchair users with spinal cord injury or lower limb amputation: Perspectives of rehabilitation professionals and wheelchair users
Source: PLoS One. 2020 Jan 31;15(1):e0228465. doi: 10.1371/journal.pone.0228465 (PMC6993975; doi:10.1371/journal.pone.0228465)
Supplement: S1 Table — (DOCX) [file pone.0228465.s003.docx]

| **S1 Table. Overview of the determinants of dietary behaviour in wheelchair users with SCI or LLA discussed by wheelchair users and rehabilitation professionals** | | | |
| --- | --- | --- | --- |
|  | **Wheelchair users (n=25)** | **Professionals (n=11)** |  |
| **Body, functions & structures** |  |  |  |
| Impairments of hand function |  | X |  |
| Impairments of digestive functions | X |  |  |
| **Personal factors: barriers** |  |  |  |
| Knowledge | X | X |  |
| Lack of intrinsic motivation |  | X |  |
| Boredom | X | X |  |
| Fatigue/lack of energy | X | X |  |
| Stage of life | X | X |  |
| Habits | X | X |  |
| Appetite | X | X |  |
| Lack of self-control | X |  |  |
| Multiple unhealthy lifestyle behaviours |  | X |  |
| **Personal factors: facilitators** |  |  |  |
| Intrinsic motivation | X | X |  |
| Goal setting | X | X |  |
| Monitoring | X | X |  |
| Risk perception | X | X |  |
| Positive experiences | X | X |  |
| Suffering |  | X |  |
| Action planning |  | X |  |
| **Health condition** |  |  |  |
| Under- and overweight | X | X |  |
| Comorbidity | X | X |  |
| Cognitive decline (LLA*) |  | X |  |
| Psychological distress |  | X |  |
| **Attitude** | X | X |  |
| **Self-efficacy** |  | X |  |
| **Environmental factors: barriers** |  |  |  |
| Not appropriately adjusted kitchens | X |  |  |
| Difficulties in measuring body weight | X |  |  |
| Eating out | X |  |  |
| Costs of healthy food | X |  |  |
| Unfavourable food supply | X |  |  |
| **Environmental factors: facilitators** |  |  |  |
| Nutrition education/counselling | X | X |  |
| Access to easy to make, healthy recipes | X |  |  |
| Knowledge and awareness of social environment | X | X |  |
| **Social influence** |  |  |  |
| Support of partners, family members or friends | X | X |  |
| Positive reinforcement by rehabilitation professionals, family and friends | X |  |  |
| Uncooperative family members | X | X |  |
| Eating with others | X |  |  |
| Cooking for others | X |  |  |
| *LLA = lower limb amputation; SCI = spinal cord injury. | | | |
